# Generation and analysis of expressed sequence tags from six developing xylem libraries in *Pinus radiata *D. Don

**DOI:** 10.1186/1471-2164-10-41

**Published:** 2009-01-21

**Authors:** Xinguo Li, Harry X Wu, Shannon K Dillon, Simon G Southerton

**Affiliations:** 1 CSIRO Plant Industry, GPO Box 1600, Canberra, ACT 2601, Australia

## Abstract

**Background:**

Wood is a major renewable natural resource for the timber, fibre and bioenergy industry. *Pinus radiata *D. Don is the most important commercial plantation tree species in Australia and several other countries; however, genomic resources for this species are very limited in public databases. Our primary objective was to sequence a large number of expressed sequence tags (ESTs) from genes involved in wood formation in radiata pine.

**Results:**

Six developing xylem cDNA libraries were constructed from earlywood and latewood tissues sampled at juvenile (7 yrs), transition (11 yrs) and mature (30 yrs) ages, respectively. These xylem tissues represent six typical development stages in a rotation period of radiata pine. A total of 6,389 high quality ESTs were collected from 5,952 cDNA clones. Assembly of 5,952 ESTs from 5' end sequences generated 3,304 unigenes including 952 contigs and 2,352 singletons. About 97.0% of the 5,952 ESTs and 96.1% of the unigenes have matches in the UniProt and TIGR databases. Of the 3,174 unigenes with matches, 42.9% were not assigned GO (Gene Ontology) terms and their functions are unknown or unclassified. More than half (52.1%) of the 5,952 ESTs have matches in the Pfam database and represent 772 known protein families. About 18.0% of the 5,952 ESTs matched cell wall related genes in the MAIZEWALL database, representing all 18 categories, 91 of all 174 families and possibly 557 genes. Fifteen cell wall-related genes are ranked in the 30 most abundant genes, including *CesA*, *tubulin*, *AGP*, *SAMS*, *actin*, *laccase, CCoAMT, MetE*, *phytocyanin, pectate lyase*, *cellulase, SuSy*, *expansin*, *chitinase *and *UDP-glucose dehydrogenase*. Based on the PlantTFDB database 41 of the 64 transcription factor families in the poplar genome were identified as being involved in radiata pine wood formation. Comparative analysis of GO term abundance revealed a distinct transcriptome in juvenile earlywood formation compared to other stages of wood development.

**Conclusion:**

The first large scale genomic resource in radiata pine was generated from six developing xylem cDNA libraries. Cell wall-related genes and transcription factors were identified. Juvenile earlywood has a distinct transcriptome, which is likely to contribute to the undesirable properties of juvenile wood in radiata pine. The publicly available resource of radiata pine will also be valuable for gene function studies and comparative genomics in forest trees.

## Background

Radiata pine (*Pinus radiata *D. Don) is the dominant forest plantation species for the sawmill industry in Australia, New Zealand, Chile and some other countries. Breeding programs in radiata pine have been conducted in Australia since the early 1950s. The first generation of breeding increased the growth rate by 33%, thus reduced the rotation period to 27–30 yrs from the previous 40–45 yrs [1]. Consequently, the faster growth rate resulted in a large proportion (30–50%) of juvenile wood in the harvested logs [2,3]. Juvenile wood has a number of undesirable wood properties [1,3] and its higher proportion in the harvested logs reduces the value of timber products. Improving juvenile wood quality and reducing its proportion have been identified as the priorities in the next generation breeding program of radiata pine. Understanding wood formation at the molecular level would underpin more efficient breeding strategies for the improvement of juvenile wood.

Genomics approaches have been applied to explore the molecular basis of growth and development in forest tree species. Expressed sequence tags (ESTs) and microarray gene expression studies have been carried out in poplar, loblolly pine, spruce and eucalypts [4-15]. Due to the economic value of wood, all forest genomic projects were primarily focused on the transcriptional regulation of wood formation (xylogenesis). Xylogenesis is initiated in the vascular cambium and proceeded from cell division to expansion, secondary wall formation, lignification, and finally programmed cell death [5,12]. Large numbers of xylogenesis ESTs from forest tree species have been deposited in public databases, including 59,797 ESTs from loblolly pine, 25,218 ESTs from poplars, 16,430 ESTs from white spruce and 52,330 ESTs from sitka spruce (extracted from [6,8,14,16], respectively). Furthermore, the genome of *Populus trichocarpa *(~550 Mbp) has been published [17], and efforts to sequence the *Eucalyptus grandis *genome (~640 Mbp) are in progress [18]. However, the large conifer genome (~20,000 Mbp) [19] is unlikely to be sequenced in the near future, thus EST sequencing remains an important approach for gene discovery in conifers.

Despite the commercial importance of radiata pine in many countries, little genomic research has been done for this species compared to loblolly pine, *Populus*, spruce, maritime pine and eucalypts. As of January 20, 2009, only 151 radiata pine ESTs appear in the NCBI GeneBank (dbEST), and no unigene information is available. Recently, 455 genes were observed to be differentially expressed in the base to the crown of the radiata pine trees using modified differential display [20]. Gene expression in the early embryogenesis of *Pinus radiata *was studied using the cDNA-AFLPs strategy, which revealed a total of 82 up- or down-regulated transcript-derived fragments (TDFs) [21]. Development of juvenile and mature wood in radiata pine is still poorly characterised at the genomics level.

We applied genomics approaches to investigate the transcriptional regulation of xylogenesis in radiata pine, with a focus on juvenile wood formation. Six developing xylem cDNA libraries were constructed from earlywood and latewood tissues collected from juvenile (7 yrs), transition (11 yrs) and mature (30 yrs) trees, respectively. The sampled xylem tissues represent the major stages of wood development in a typical rotation period of radiata pine. A total of 6,389 high quality xylogenesis ESTs were sequenced from 5,952 cDNA clones and assembled into 3,304 unigenes. Here we report the generation and analysis of a genomic resource for wood formation in radiata pine.

## Results

### EST sequencing and assembly

In total, 6,389 high quality ESTs of at least 100 bp in length were collected from approximately 8,000 raw sequences. The 6,389 ESTs were sequenced from 5,952 different cDNA clones in six developing xylem cDNA libraries. Average size of all 6,389 ESTs and the 5,952 ESTs from 5' end sequences is 624 bp and 636 bp, respectively. Of the 5,952 ESTs, 86.4% and 69.6% are greater than 300 bp and 500 bp in length, respectively. The number of ESTs in each library ranged from 694 in earlywood at transition age to 1,636 in latewood at juvenile age (Table 1). Juvenile wood has the highest proportion of ESTs due to the focus of this study. The assembly of all 5,952 ESTs from 5' end sequences generated 3,304 xylogenesis unigenes, including 952 contigs (28.8%) and 2,352 singletons (71.2%) (Table 1).

**Table 1:** Assembly of radiata pine xylogenesis ESTs from six cDNA libraries

*Assembly*	*EST*	*Contig*	*Singleton*	*Unigene*	*Redundancy (%) *^*c*^
Assembly for six libraries ^a^	5,952	952	2,352	3,304	47.8
Assembly for each library ^b^					
Juvenile earlywood	1,259	198	711	909	27.8
Juvenile latewood	1,636	241	935	1,176	28.1
Transition earlywood	694	92	410	502	27.7
Transition latewood	799	73	371	444	44.4
Mature earlywood	837	128	454	582	30.5
Mature latewood	727	65	559	624	14.2
Total of each library	5,952	797	3,440	4,237	28.8 ^d^

^a^: ESTs from the 5 ends of 5,952 clones were used in the assembly;

^b^: Only 5 end ESTs in each library were used in the assemblies;

^c^: Redundancy was estimated by: 1- (number of unigenes/number of ESTs);

^d^: The average redundancy from each of six cDNA libraries.

**Figure 1:**
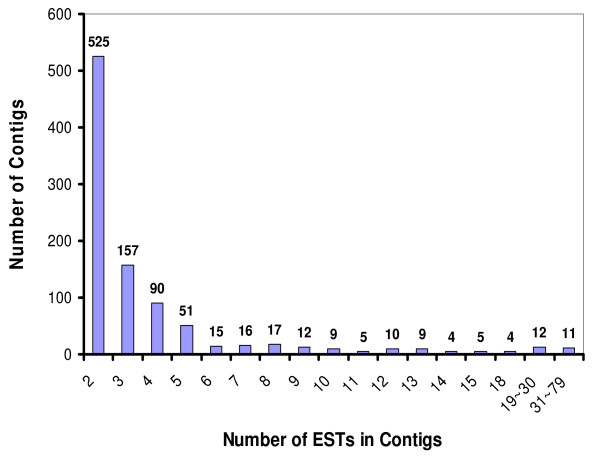
**Distribution of 952 contigs based on the number of clustered ESTs**. The number of ESTs clustered in contigs reduces the efficiency of ESTs for representing different genes, thus can be regarded as an indicator for EST redundancy in cDNA library.

The 3,304 unigenes have an average length of 702 bp; 41.0% are more than 800 bp, and only 13.5% are less than 300 bp. Of the 952 contigs, 28.4% (270) have four or more transcripts (Figure 1) and the three deepest contigs included 69–79 transcripts. Since the ESTs and unigenes were derived from a total of seven different genotypes of radiata pine, these deep contigs provide an opportunity to identify single nucleotide polymorphisms (SNPs). The number of ESTs forming a contig reflects the level of EST redundancy in the cDNA libraries. The assembly of 5,952 ESTs from 5' end sequences revealed 47.8% of redundancy in the radiata pine EST collection (Table 1). However, the EST redundancy in each library varies from 14.2% to 44.4%, with an average redundancy of 28.8% (Table 1). The redundancy may increase significantly during further EST sequencing. By comparison, the EST redundancy in the radiata pine EST collection is slightly higher than the estimated redundancy of 28% in *Populus *(13.5 k microarrays) [22] and of 39% in white spruce (50,000 ESTs) [14].

### Functional annotation and classification

Blast searches of the 5,952 ESTs against the NCBI nr database revealed 73.4% and 69.0% matches with tblastx and blastx (E-value ≤ 10^-5^), respectively. However, 71.3% of all matches with blastx are either unknowns (47.5%), unnamed (13.6%), hypothetical (5.4%) or predicted proteins (4.7%). When using the UniProt database with blastx (E-value ≤ 10^-5^) and TIGR database with blastn (E-value ≤ 10^-15^), 97.6% of the 5,952 ESTs have homologs and only 20.5% of all matches are unknowns or uncharacterized proteins. Of the 139 ESTs (2.3%) with no matches in the UniProt and TIGR databases, 41 ESTs are greater than 500 bp in length and remain as singletons after assembly, thus some of which are likely to represent putative novel ESTs in radiata pine wood formation. Based on the Pfam known protein family database, 52.1% of the ESTs have homologs with blastx (E-value ≤ 10^-5^) and were classified into 772 protein families. Nearly half of the ESTs (47.9%) did not match the known protein families in the Pfam database, thus they were regarded as unknown protein families.

**Figure 2:**
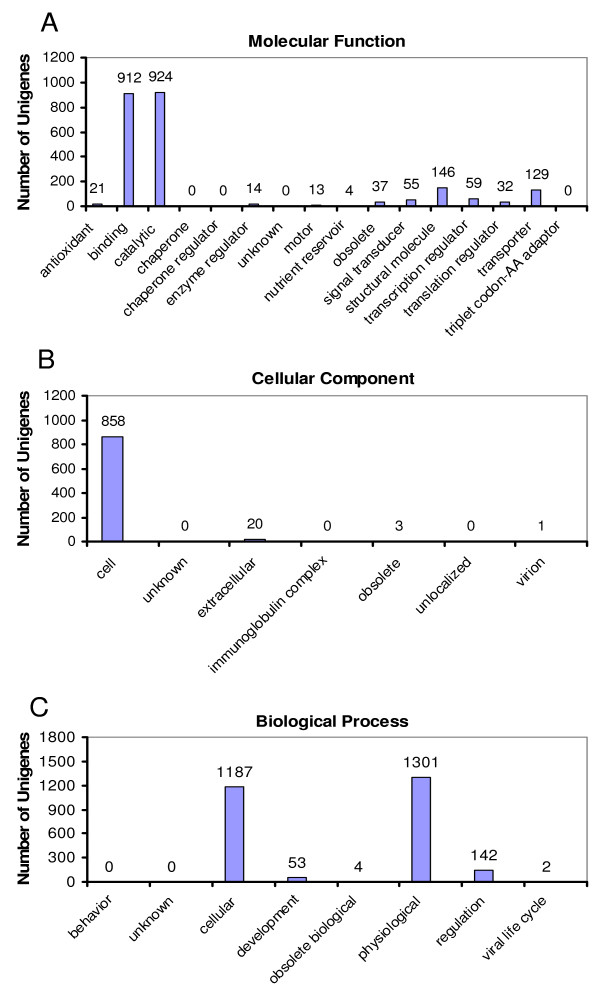
**Functional classification for the 1,843 unigenes which were assigned with GO terms**. Among 3,304 unigenes from radiata pine xylogenesis, 1,813 unigenes were functionally assigned with GO terms as molecular function (A), cellular component (B) and biological process (C). For each of these GO terms, functions were further assigned with sub-category of GO terms. The total percentage of functional categories or sub-category may be over 100% due to possibly multiple functions from some unigenes.

Of the 3,304 xylogenesis unigenes, 68.1% have matches in the NCBI nr database with blastx, however 77.3% of all matches are unknowns or uncharacterized proteins. In contrast, a total of 96.1% of the unigenes matched sequences in the UniProt (with blastx) and TIGR (with blastn) databases and only 42.9% of all matches were not assigned GO terms (Table 2). The results blasted with unigenes are similar to those with ESTs. In the functional classification with GO terms, 89.1% of the 1,813 unigenes with assigned GO terms have molecular functions, 74.6% are involved in a biological process, and 47.8% are cellular components. The three categories of GO terms fell predominantly into one or two sub-categories (Figure 2). In the molecular function category with 1,616 unigenes 56.4% and 57.2% have binding and catalytic activity, respectively. Of the 867 unigenes in the cellular component, 98.9% are related to cell components. As for the 1,353 unigenes involved in biological process, 87.7% and 96.2% have functions in cellular process and physiological process, respectively.

**Table 2:** Summary of annotation and functional classification of 3,304 xylogenesis unigenes in radiata pine*.

	*No*.	*Hits*	*No Hits*	*With GO Terms*	*Cellular Component*	*Molecular Function*	*Biological Process*	*No GO terms*
Contig	952	942 (98.9%)	10 (1.1%)	593 (62.3%)	272 (28.6%)	524 (55.0%)	453 (47.6%)	349 (36.7%)
Singleton	2,352	2,232 (94.9%)	120 (5.1%)	1,220 (51.9%)	595 (25.3%)	1,092 (46.4%)	900 (38.3%)	1,012 (43.0%)
Unigene	3,304	3,174 (96.1%)	130 (3.9%)	1,813 (54.9%)	867 (26.2%)	1,616 (48.9%)	1,353 (41.0%)	1,361 (41.2%)

*: Annotation was based on blastx with unigenes against UniProt; for the no hits a further blastn was performed against the TIGR database; functional classification was based on GO terms.

The functional annotation of 3,304 unigenes using the UniProt database revealed 2,101 (63.6%) matches, which is slightly lower than the matches using the NCBI nr database. Of the 2,101 matched unigenes 1,813 were assigned with GO terms and matched 1,582 different UniProt accession numbers, which suggests that possibly 1,582 non-redundant genes with known functions have been identified in wood formation of radiata pine. Significant GO term enrichments of the xylogenesis related genes in radiata pine (Table 3) were revealed using the DAVID Bioinformatics Resources 2008 [23,24]. The mostly enriched GO terms in biological process (BP) include cellular protein metabolic process, transport, cellular component organization and biogenesis, and catabolic process. In cellular component (CC), cytoplasmic part, macromolecular complex, and non-membrane-bound organelle are the mostly enriched terms. While in the molecular function (MF), nucleotide binding, transporter activity, peptidase activity, and structural molecular activity are highly enriched. Further DAVID functional classification revealed 13 functional groups with an enrichment score of at least 1.26 (Additional file 1). The most significant functional group is a cluster of 11 genes showing a peptidase function.

**Table 3:** The most enriched GO terms in the radiata pine xylogenesis genomic resource*.

*Category*	*GO Term*	*Genes*	*%*	*P-value*
BP	GO:0044267 cellular protein metabolic process	79	4.99	0.025
BP	GO:0006810 transport	47	2.97	0.006
BP	GO:0016043 cellular component organization and biogenesis	36	2.28	0.011
BP	GO:0009056 catabolic process	17	1.07	0.060
CC	GO:0044444 cytoplasmic part	99	6.26	0.000
CC	GO:0032991 macromolecular complex	46	2.91	0.001
CC	GO:0043228 non-membrane-bound organelle	23	1.45	0.074
MF	GO:0000166 nucleotide binding	55	3.48	0.088
MF	GO:0005215 transporter activity	25	1.58	0.059
MF	GO:0008233 peptidase activity	19	1.20	0.014
MF	GO:0005198 structural molecule activity	17	1.07	0.002

* A total of 1,582 unigenes with homologs and non-redundant accession numbers in UniProt are included in the DAVID functional annotation analysis, only the enriched GO terms including more than 1% of genes are listed.

### Cell wall biosynthesis genes

In the 5,952 ESTs, only 6.8% have homologs (blastx, E-value ≤ 10^-5^) in the Cell Wall Navigator, a primary wall gene database of *Arabidopsis *[25]. However, all 18 categories of primary and secondary wall genes in the MAIZEWALL database [26] were represented in the radiata pine EST resource, including 1,070 ESTs classified into 91 cell wall gene families (Additional file 2). Therefore, genes related to secondary cell walls are highly accumulated in the radiata pine EST resource. The 1,070 cell wall related ESTs of radiata pine were previously assembled into 826 contigs and 19 singletons, which matched sequences in the UniProt and TIGR databases with 557 non-redundant accession numbers, suggesting possibly 557 cell wall-related genes occurred in the radiata pine EST resource. The most abundant cell wall gene is *cellulose synthase *(*CesA*), with a total of 175 ESTs (2.9%). Other cell wall related genes in the 30 most highly abundant genes include *tubulin*, *arabinogalactan protein *genes (*AGP*), *S-adenosylmethionine synthetase *(*SAMS*), *actin*, *laccase, CCoAMT, methionine synthase *(*cobalamin-independent*) (*MetE*),*phytocyanin, pectate lyase*,*cellulase, sucrose synthase *(*SuSy*), *expansin*, *chitinase *and *UDP-glucose dehydrogenase *(Table 4).

**Table 4:** Thirty highly abundant genes (or gene families) in the 5,952 xylogenesis ESTs of radiata pine.

*Gene or gene family*	*ESTs*	*%*
*Cellulose synthase *(*CesA*)	175	2.94
*Ribosomal protein*	165	2.77
*Tubulin *(*TUB*)	102	1.71
*Aquaporin*	102	1.71
*Arabinogalactan protein *(*AGP*)	89	1.50
*Phytochrome*	75	1.26
*Actin*	58	0.97
*S-adenosylmethionine synthetase *(*SAMS*)	46	0.77
*Methionine synthase *(*cobalamin-independent*)(*MetE*)	41	0.69
*Elongation factor*	38	0.64
*Photoassimilate-responsive protein *(*PAR*)	38	0.64
*Laccase*	36	0.60
*Pectate lyase*	35	0.59
*Auxin-induced protein*	32	0.54
*Caffeoyl-CoA O-methyltransferase *(*CCoAMT*)	29	0.49
*Unknown *(*Emb| CAB86899.1*)	29	0.49
*Endo-1,4-beta-D-glucanase *(*cellulase*)	29	0.49
*Unknown *(*Os07g0462200*)	29	0.49
*Phytocyanin*	29	0.49
*Ubiquitin*	28	0.47
*Cytokinin-binding protein*	27	0.45
*Sucrose synthase *(*SuSy*)	26	0.44
*Eukaryotic translation initiation factor*	25	0.42
*Zinc finger*	25	0.42
*Chitinase*	24	0.40
*RNA-binding protein*	24	0.40
*Metallothionein-like protein class II *(*MT-II*)	23	0.39
*Pollen-specific protein C13*	23	0.39
*Expansin*	21	0.35
*UDP-glucose dehydrogenase*	21	0.35

Some cell wall genes were moderately abundant (10–20 ESTs) in the radiata pine EST resource, including *protective protein for beta-galactosidase *(*PPGB*), *methionine synthase*, *proline-rich protein *(*PRP*), *translationally controlled tumor protein *(*TCTP*), *alpha-galactosidase*, *UDP-glucose glucosyltransferase*, *malate dehydrogenase*, *pectinesterase*, and *glycine-rich protein *(*GRP*). Other cell wall related genes including *pectin methylesterases *(*PMEs*) and *xyloglucan endotransglycosylases *(*XETs*) have five ESTs each. Eight ESTs encoding cyclin or cyclin-like and seven ESTs encoding cell cycle proteins were identified in radiata pine. These cell cycle genes can activate cell-cycle machinery and cell division [27]. Interestingly, most genes involved in lignin biosynthesis were present in radiata pine wood formation. *Laccases*, *SAMS *and *CCoAOMT *are in the 30 highly abundant genes with 29 to 46 ESTs (Table 4). *C4H*, *4CL*, *peroxidase*, *CAD*, *C3H*, *COMT *and *dirigent-like protein *are moderately abundant with seven to 18 ESTs. *CCR *and *PAL *also occurred with two ESTs each in the radiata pine EST resource.

Based on the Pfam database, 772 known protein families were identified in radiata pine. The 30 most highly abundant families included 11 protein families related to cell wall biosynthesis (Table 5). Unsurprisingly, cellulose_synt is the most abundant family with 108 ESTs. Other abundant protein families included actin, tubulin, tubulin_C, meth_synt_2, methltransf_3, peroxidase, S-AdoMet_synt_C, glyco_hydro_19 and glyco_hydro_9 (Table 5). These abundant families included many known proteins associated with cell wall formation, such as CesAs, actins, tubulins (α and β), methionine synthases, MetE, CCoAOMT, peroxidases, SAMS, chitinases and cellulases. However, AGPs are not recognized as proteins in the Pfam database, thus they are not listed in the abundant protein families. The most abundant protein families here are broadly consistent with the most abundant genes based on UniProt and TIGR databases (Table 4).

**Table 5:** Thirty highly abundant protein families in the radiata pine xylogenesis EST resource.

*Protein family*	*Function annotation*	*Pfam accession*	*ESTs*	*%*
Cellulose_synt	Cellulose synthase	PF03552	108	1.81
MIP	Major intrinsic protein	PF00230	90	1.51
Pkinase	Protein kinase domian	PF00069	58	0.97
Actin	Actin	PF00022	50	0.84
Tubulin_C	Tubulin/FtsZ family, C-terminal domain	PF03953	45	0.76
Cu_bind_like	Plastocyanin-like domain	PF02298	41	0.69
P450	Cytochrome P450	PF00067	38	0.64
Uiquitin	Ubiquitin family	PF00240	38	0.64
S-AdoMet_synt_M	S-adenosylmethionine synthetase, Central domain	PF02772	37	0.62
RRM_1	RNA recognition motif	PF00076	35	0.59
Meth_synt_2	Cobalamin-independent synthase, Catalytic domain	PF01717	35	0.59
DUF1218	Unknown function protein	PF06749	34	0.57
Methyltransf_3	O-methyltransferases	PF01596	30	0.50
PAR1	Photoassimilate-responding protein	PF06521	30	0.50
Ras	Ras superfamily	PF00071	28	0.47
Hin1	Harpin-induced protein 1 (Hin1)	PF07320	27	0.45
Tryp_alpha_amyl	Protease inhibitor/seed storage/LTP family	PF00234	26	0.44
Tubulin	Tubulin/FtsZ family, GTPases domain	PF00091	25	0.42
Cu-oxidase_2	Multicopper oxidase-like domains	PF07731	25	0.42
Peroxidase	Peroxidase	PF00141	24	0.40
Efhand	Signaling proteins and buffering/transport proteins	PF00036	23	0.39
Mito_carr	Mitochondrial carrier protein	PF00153	23	0.39
UQ_con	Ubiquitin-conjugating enzyme	PF00179	23	0.39
Peptidase_C1	Papain family cysteine protease	PF00112	21	0.35
Glyco_hydro_9	Glycosyl hydrolase family 9	PF00759	21	0.35
Arf	ADP ribosylation factor (Arf)	PF00025	20	0.34
Thioredoxin	Thioredoxin	PF00085	20	0.34
Ricin_B_lectin	Ricin-type beta-trefoil lectin domain	PF00652	20	0.34
Glyco_hydro_19	Chitinase class I	PF00182	19	0.32
Epimerase	NAD dependent epimerase/dehydratase family	PF01370	19	0.32

### Identification of transcription factors

PlantTFDB, a recently developed database of transcription factor (TF) families for 22 plant species [28], was used to identify putative transcription factors expressed in radiata pine wood formation. Blastx searches revealed 358 ESTs (assembled into 284 unigenes) of radiata pine with matches in PlantTFDB at E-value ≤ 10^-5^. These homologs fell into 41 families (Table 6) and represented 64.1% of the 64 TF families in the poplar genome, suggesting extensive involvement of transcription factors in the regulation of xylogenesis gene expression. The most abundant TF family in radiata pine wood formation is PHD (Cys4-His-Cys3 zinc finger) with 55 ESTs. Other TF families with at least five ESTs included C3H, HB, C2H2, NAC, MYB-related, MYB, AP2-EREBP, PcG, bHLH, LIM, EIL, HMG, C2C2-GATA, LUG, WRKY, bZIP, GARP-G2-like, SBP and Trihelix. The higher abundance of PHD, C3H, MYB, HMG, WRKY, NAC, HB and bZIP in radiata pine xylogenesis was also observed in white spruce [14].

**Table 6:** Forty-one transcription factor families in the radiata pine xylogenesis EST resource.

*TF family*	*Description*	*ESTs*	% *
PHD	Cys4--His--Cys3 zinc finger	55	15.36
C3H	Zinc finger, C-x8-C-x5-C-x3-H type (and similar)	38	10.61
HB	Homeobox domain	29	8.10
C2H2	Zinc finger, C2H2 type	23	6.42
NAC	No apical meristem (NAM) protein	22	6.15
MYB-related	N-terminal myb-domain	20	5.59
MYB	Myb-like DNA-binding domain	16	4.47
AP2-EREBP	AP2 domain	15	4.19
PcG	Polycomb group (PcG) proteins	14	3.91
bHLH	Helix-loop-helix DNA-binding domain	11	3.07
LIM	LIM domain	11	3.07
EIL	Ethylene insensitive1 (EIN3)	10	2.79
HMG	HMG (high mobility group) box	8	2.23
C2C2-GATA	GATA zinc finger	7	1.96
LUG	LEUNIG gene	7	1.96
WRKY	WRKY DNA-binding domain	7	1.96
bZIP	Basic leucine zipper (bZIP) motif	6	1.68
GARP-G2-like	GLK proteins	5	1.40
SBP	SBP-domain	5	1.40
Trihelix	Trihelix DNA-binding domain	5	1.40
MADS	DNA-binding and dimerisation domain	4	1.12
CCAAT-Dr1	CCAAT-box-related motifs	4	1.12
ZIM	Zinc-finger protein expressed in inflorescence meristem	4	1.12
Alfin	Cys4 zinc finger and His/Cys3	3	0.84
AUX-IAA	AUX/IAA family	3	0.84
TAZ	TAZ zinc finger	3	0.84
ABI3-VP1	ABI3/VP1 proteins	2	0.56
AS2	Asymmetric leaves2	2	0.56
BES1	BRI1-EMS-Suppressor 1	2	0.56
C2C2-CO-like	CCT motif	2	0.56
CAMTA	Calmodulin-binding transcription activators	2	0.56
FHA	Forkhead domain	2	0.56
GRF	Growth-regulating factor1	2	0.56
MBF1	Multiprotein bridging factor 1	2	0.56
ARF	Auxin response factor	1	0.28
ARID	AT-rich interaction domain	1	0.28
CCAAT-HAP3	CCAAT-binding factor	1	0.28
HSF	Heat shock factor	1	0.28
Nin-like	Nodule inception protein	1	0.28
TLP	Tubby family	1	0.28
ZF-HD	ZF-HD class homeobox domain	1	0.28

*: % is based on the 358 radiata pine ESTs with homologs in the PlantTFDB.

### Transcriptome reorganization during wood development

**Figure 3:**
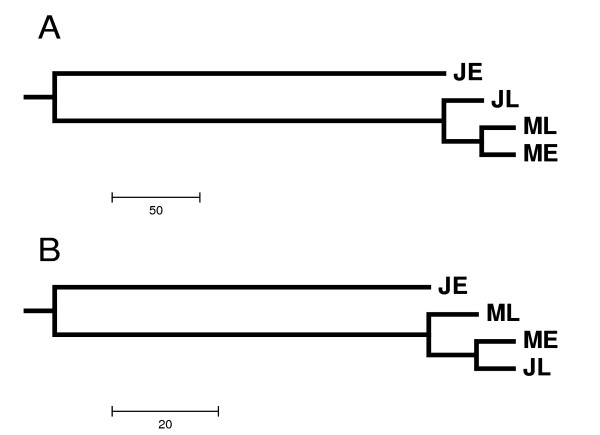
**Hierarchical clustering dendrogram trees for juvenile and mature wood cDNA libraries**. ESTs or unigenes from each of the four libraries (juvenile earlywood-JE, juvenile latewood-JL, mature earlywood-ME and mature latewood-ML) were annotated against the UniProt and TIGR databases, and assigned with GO terms. The abundance of ESTs or unigenes in each GO term was calculated for each library, and further normalized as a percentage based on the number of ESTs or unigenes in each library. Statistical significance of EST or unigene abundance in each GO term among the four libraries was valuated by Chi Square tests and P-values. The normalized EST or unigene abundance was used to construct the hierarchical clustering dendrogram trees. A: the dendrogram tree for ESTs. B: the dendrogram tree for unigenes.

The ESTs and unigenes from different libraries represent the transcriptome in the respective wood development stages. We compared the transcriptomes from juvenile earlywood, juvenile latewood, mature earlywood and mature latewood tissues. These four tissues were collected from trees growing within 50 m of each other, and at the same date for earlywood or latewood to minimize the environmental effects. Comparative transcriptome analysis highlighted 306 and 150 GO terms showing significant differences (P-value < 0.01) in terms of EST and unigene abundance, respectively. The hierarchical clustering dendrogram trees from both ESTs and unigenes revealed similar patterns of transcriptome reorganization during wood formation in radiata pine (Figure 3A, B), which suggested that juvenile earlywood has a distinct transcriptome compared to the other three developmental stages, whereas the transcriptome of juvenile latewood is more conserved with mature earlywood and latewood. The distinct gene expression in juvenile earlywood is likely associated with the unique properties of juvenile wood.

Comparative analysis of protein family revealed some known protein families more abundant in particular libraries (Additional file 3). Pectinesterase, pfkB family carbohydrate kinase, UDP-glucose/GDP-mannose dehydrogenase, homeobox domain, UDP-glucosyl transferase, profilin, thioredoxin, and plastocyanin-like domain were more abundant in earlywood. In contrast, CesA, SAMS, TCTP, tubulin, dehydrin, metallothionein, protein tyrosine kinase, aminotransferase, and WD domain protein were more abundant in latewood. The protein families more abundant in juvenile wood include pectate lyase, skp1, zinc-binding dehydrogenase and inorganic H+ pyrophosphatase. While plastocyanin-like, peroxidase, elongation factor, calreticulin, UDP-glucose/GDP-mannose dehydrogenase, SAMS, glycosyl hydrolases family 17, aldehyde dehydrogenase, major intrinsic protein (MIP), pectinesterase, CesA and O-methyltransferase are more abundant in mature wood. Some protein families are highly represented in both latewood and mature wood, suggesting possibly similar transcriptional regulation in these developmental stages.

Genes or gene families with more expression in different libraries were also identified in the radiata pine EST resource (Additional file 4). *Homeodomain*, *AGP4*, *LTP*, *peroxidase*, *glycosyl transferase*, *actin 2*, *PRP*, *malate dehydrogenase*, *phytocyanin *and *PPGB *have higher expression in earlywood. In contrast, *expansin *(*ripening-related*), *dehydrin*, *tubulin alpha 1*, *COMT*,*CC-NBS-LRR resistance-like*, and *green ripe-like 1 *are more abundant in latewood. Genes more expressed in juvenile wood include *methionine synthase*, *HB1*, *cytochrome c oxidase*, *alpha tubulin 1*, *metallothionein-like*, *AGP6*, etc. Some genes related to secondary wall biosynthesis (ie, *peroxidase*, *AGP5*, *FLA17*, *CAD *and *CesA1*) are more expressed in mature wood. Interestingly, in the large *AGP *gene family, *PrAGP4 *is more expressed in earlywood, while *PrAGP5 *and *PrFLA17 *are more expressed in mature wood, suggesting divergent roles of different *AGP *genes during wood formation.

GO term enrichments in different stages of wood development were also revealed using DAVID Bioinformatics Resources 2008 (Additional file 5). Specifically enriched GO terms in earlywood included: cellular component organization and biogenesis, membrane, protein complex, catalytic activity, hydrolase activity and transporter activity. Latewood specifically enriched terms included: cellular macromolecule metabolic process, protein modification process, cytoplasm, nucleotide binding, and protein kinase activity. The number of specifically enriched GO terms in juvenile wood is about two times as in mature wood, suggesting more unique gene expression in juvenile wood than mature wood (Additional file 5). The most enriched specific GO terms in juvenile wood included: cellular macromolecule metabolic process, intracellular organelle part, protein complex, hydrolase activity, and nucleotide binding; while in latewood cellular component organization and biogenesis, membrane, and transporter activity are the most enriched specific GO terms. The specifically enriched GO terms in different development stages are likely to reflect their unique transcriptomes.

## Discussion

The 6,389 xylogenesis ESTs and 3,304 unigenes described here form a significant genomic resource for radiata pine. Being derived from major stages of wood development in a typical rotation period, this resource represents a broad xylogenesis transcriptome in the species. The highest proportion of ESTs is derived from juvenile wood and will assist in understanding transcriptional regulation at this stage. Most families of genes involved in secondary cell wall development are represented in the EST resource.

### Gene expression in secondary wall formation

Lignin biosynthesis is a key developmental feature that distinguishes secondary from primary cell walls. The synthesized monolignols are transported to the apoplast, where polymerisation starts in the middle lamella and cell corners [29]. Most genes in the monolignol pathway are represented with high or moderate abundance in radiata pine xylogenesis including *SAMS*, *MetE*,*laccase, CCoAOMT*, *C4H, 4CL*,*methionine synthase*,*peroxidase*,*CAD, C3H*, *COMT *and *cytochrome c oxidase*. A total of 46 radiata pine ESTs (four contigs and two singletons) were found to encode SAMS protein. Lignin precursors are extensively methylated in the S-adenosyl methionine (SAM) dependent reaction [30]. In white spruce seven *SAMS *genes were previously identified [14]. Some other lignin biosynthetic genes were also identified at lower abundance including *CCR*, *PAL *and *dirigent-like*. The dirigent protein was presumed to act as a template for lignin polymerization [31]. The presence and abundance of most lignin biosynthetic genes in the radiata pine EST collection suggests active secondary cell wall biosynthesis in the xylem tissues sampled.

**Figure 4:**
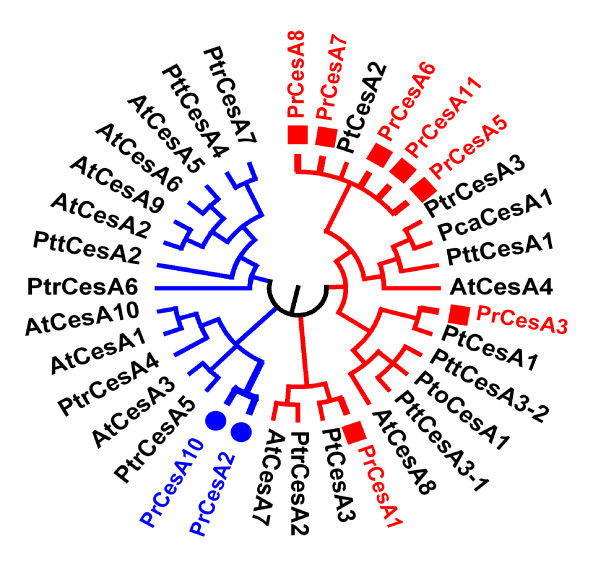
**Phylogenetic tree of CesA proteins from radiata pine and other species**. Muscle software was used to align multiple protein sequences of CesAs derived from GenBank. The protein sequences are deduced amino acid sequences from full length cDNAs except for some radiata pine CesAs. GenBank accession numbers are as follows: *Pinus radiata*, PrCesA1 (AAT57672), PrCesA2 (AAQ63936), PrCesA3 (AAQ63930), PrCesA5 (AAQ63931), PrCesA6 (AAQ63932), PrCesA7 (AAQ63933), PrCesA8 (AAQ63934), PrCesA10 (AAQ63935), PrCesA11 (AAQ63929). PrCesA1 and PrCesA10 are full length sequences, PrCesA2 is nearly full length sequence, others are partial sequences; *Pinus taeda*, PtCesA1 (AAX18647), PtCesA2 (AAX18648), PtCesA3 (AAX18649); *Populus tremuloides*, PtrCesA2 (AAM26299), PtrCesA3 (AAQ08987), PtrCesA4 (AAO25536), PtrCesA5 (AAL23710), PtrCesA6 (AAP40636), PtrCesA7 (AAO25581); *Populus tremula × Populus tremuloides*, PttCesA1 (AAT09894), PttCesA2 (AAT09895), PttCesA3-1 (AAT09896), PttCesA3-2 (AAT09897); *Populus tomentosa*, PtoCesA1 (AAY21910); *Populus canescens*, PcaCesA1 (AAC78476); *Arabidopsis thaliana*, AtCesA1 (O48946), AtCesA2 (O48947), AtCesA3 (Q941L0), AtCesA4 (Q84JA6), AtCesA5 (Q8L778), AtCesA6 (Q94JQ6), AtCesA7 (Q9SWW6), AtCesA8 (Q8LPK5), AtCesA9 (Q9SJ22), AtCesA10 (Q9SKJ5). Red colour indicates secondary cell wall CesAs, blue colour indicates primary cell wall CesAs.

Cellulose is deposited in both primary and secondary cell walls, but is much more abundant in secondary walls. *CesA *is the most abundant gene in the radiata pine EST collection with 175 ESTs representing six of the eight previously isolated *PrCesA *genes [32]. A phylogenetic tree of plant CesAs (Figure 4) constructed using Muscle [33] and MEGA 4 [34] revealed that five of the six PrCesA proteins identified in this study are most homologous to the secondary wall CesAs of *Arabidopsis *[35]. The five PrCesAs are also most homologous to the secondary wall CesAs of *Populus *[36-38] and loblolly pine [39]. Interestingly, the five PrCesAs cover all three sub groups of plant secondary wall CesAs represented by three *Arabidopsis *secondary wall CesAs (AtCesA4, AtCesA7 and AtCesA8), respectively, suggesting the functional conservation of secondary wall CesAs across angiosperm and gymnosperm taxa. A single EST encoding PrCesA10 was clustered with primary cell wall CesAs, suggesting primary wall *PrCesA *genes are poorly presented in the radiata pine EST resource. *Cellulases *and *SuSy *are also highly expressed during radiata pine wood formation. Cellulase may be part of the cellulose synthesizing complex and required for wall assembly and elongation [40]. A membrane-bound cellulase isoenzyme is up-regulated in poplar secondary walls [5]. In Scots pine SuSy activity was observed to peak in the zone of maturing tracheids where the secondary wall is formed, and its expression was lowest in primary wall tissues [41].

Hemicelluloses and pectin form a large group of heteropolysaccharides composed of D-xylose, L-arabinose, L-rhamnose, L-fucose, D-mannose, D-galactose, D-galacturonate, or D-glucose. In the radiata pine EST collection we identified genes involved in the synthesis and degradation of hemicellulose and pectin biosynthesis, including 21 ESTs (three contigs) of *UDP-glucose dehydrogenase*. UDP-D-glucuronate synthesis is the rate-limiting step for the biosynthesis of both hemicellulose and pectin [42]. We also identified four radiata pine ESTs of *xyloglucan endotransglycosylases *(*XETs*). *XETs *can cut and rejoin xyloglucan (XG) chains, and are believed to be important regulators of primary cell wall expansion [43]. One radiata pine *XET *is most homologous to *Arabidopsis XTH8*, which belongs to the same class as *PttXET16A*, a poplar secondary wall *XET *[44]. The considerable involvement of *XETs *in secondary walls is likely in the breakage and reconnection of linkages between adjacent microfibrils soon after their synthesis.

*AGPs*, *AGP-like *proteins and *FLAs *are highly abundant in the radiata pine EST collection with a total of 89 ESTs. The ortholog of loblolly pine *PtaAGP4 *is the most abundant *AGP *in radiata pine with 45 ESTs. Other identified radiata pine *AGPs *include *PrAGP5*, *PrAGP6*, *PrAGP-like *and *PrFLAs *(*PrFLA1*, *8*, *10*, *12*, *16*, *17 *and *26*). Six loblolly pine *PtaAGPs *(*AGP3*, *AGP4*, *AGP5*, *AGP6*, *3H6 *and *14A9*) were predominantly expressed in xylem [45]. The role of these *AGPs *in secondary wall development is currently unknown. In loblolly pine *PtaAGP6 *epitopes are restricted to cells formed immediately before secondary cell wall thickening [46]. However, the preponderance of genes in the radiata pine EST resource that are clearly involved in secondary wall development suggests that AGPs may also play an important role in secondary wall synthesis. *AGPs *were also found to be expressed in xylem tissues of Angiosperms. In *Populus *15 *FLAs *are expressed in xylem, ten of which are up-regulated in tension wood [47]. While in eucalypts two *FLAs *are strongly up-regulated in upper branch wood [15]. A tobacco AGP is a candidate linker at the cell surface to mediate signal transduction between the plasma membrane and the cytoskeleton [48].

### Expression of cytoskeleton-related genes

Conspicuous in the radiata pine xylem EST resource are genes encoding the cytoskeletal proteins: tubulin, actin and other cytoskeletal-associated proteins. The cytoskeleton provides the structural basis for cell polarity establishment and maintenance [49,50]. Cellulose microfibril arrangement and deposition is believed to be directed by cortical microtubules, the dynamic heteropolymer arrays of α-tubulin and β-tubulin proteins. A *Eucalyptus grandis β-tubulin *gene (*EgrTUB1*) has been implicated in determining the orientation of cellulose microfibrils in plant secondary walls [51]. Two *Populus tremuliodes α-tubulin *genes (*TUA1 *and *TUA5*) are highly expressed in wood tissues [52] and ten *tubulin *genes in *Populus tremula *× *P. tremuliodes *are up-regulated in secondary walls [5]. In the radiata pine EST resource, a total of 102 ESTs were annotated to encode tubulin proteins, among which three *tubulin *genes (*PrTUA1*, *PrTUB2 *and *PrTUB3*) are highly or moderately abundant with 52, 26 and 15 ESTs, respectively. These three *PrTUBs *are most homologous to poplar *tubulin *genes that are strongly expressed in xylem tissues [52].

Actin microfilaments ensure the delivery of vesicles to specific sites in plant cells and are involved in cell shape determination [53]. *Actin *genes are highly abundant in the radiata pine EST collection with a total of 58 ESTs (six contigs and five singletons). Other actin related and interacting genes are also found, including *ADF*, *actin binding protein *(*ABP*), *actin-related protein *(*ARP*), *profilin *and *villin*. *Arabidopsis *has ten actin genes [53], eight actin-related proteins (ARP) [54] and many actin-interacting proteins including profilin, actin depolymerisation factor (ADF), fimbrin, villin, rho-type small GTPase, capping protein, and actin-interacting cyclise-associated protein [55,56]. The radiata pine *ARPs *are the putative homologs to *Arabidopsis ARP2*, *ARP3 *and *ARP6*, suggesting the possible involvement of the ARP2/3 complex in actin cytoskeleton development during radiata pine xylem formation.

Cytoskeleton formation involves cycling dynamics of polymerization and depolymerization of its basic structural subunits, such as G-actin and tubulin dimers. Microtubule-associated proteins (MAPs) and actin-binding proteins (ABPs) are believed to regulate the dynamic cytoskeletal changes [57]. In the radiata pine EST resource, several transcripts are present for MAPs (three ESTs), microtubule-binding protein (one EST) and actin-binding protein (two ESTs). Kinesin-1 (conventional kinesin) is a dimeric motor protein that carries cellular cargo along microtubules [58]. A novel kinesin (GhKCH1) from cotton fibers plays a role in coordinating the actin network with the cortical microtubule array [59]. We identified eight radiata pine ESTs annotated as kinesin-1 or kinesin-like proteins which may have similar roles in actin and microtubule interactions.

### Transcription factors in secondary wall formation

Of the 64 transcription factor families in poplar genome, 41 families (64.1%) are presented in the radiata pine xylogenesis genomic resource (Table 6). These transcription factor families include many regulatory genes, such as *MYBs*, *MADS-box*, *LIM domain, zinc finger*, *Class III HD-Zip*, *WRKY *and *AUX/IAA*. Several members of NAC, MYB, zinc finger, LIM domain, MADS-box, AUX/IAA and homeodomain (HD) are believed to regulate secondary wall biosynthesis [60-65]. MYBs and zinc fingers can recognise AC elements in the promoters of many monolignol biosynthesis genes [65-67]. Some members of the NAC and MYB families are the key switches in the transcriptional network for secondary wall development [65].

Three radiata pine ESTs of *R2R3-type MYB *are most homolgous to the three secondary xylem *MYB *genes (*MYB2*, *MYB4 *and *MYB8*) in loblolly pine and *Picea glauca *[68]. In loblolly pine *PtMYB1 *and *PtMYB4 *are transcriptional activators of lignin synthesis [66,67]. The *Eucalyptus EgMYB2 *regulates lignin biosynthesis through binding to AC element [69]. Homeobox domain (HD) transcription factors were also identified in the radiata pine EST resource with 29 ESTs (two contigs). Three radiata pine ESTs were annotated as *AUX/IAA *genes. Auxin can rapidly induce *AUX/IAA *gene expression [70] and five *AUX/IAA *genes from hybrid aspen are up-regulated in xylem [71].

In radiata pine EST resource *Class III HD-Zip *genes were represented by 15 ESTs (six unigenes). Five *Arabidopsis HD-Zip III *genes are up-regulated in secondary xylem [63,72] and the hybrid aspen *class III HD-Zip *gene (*PtaHB1*) is closely associated with wood formation [73]. Eleven radiata pine ESTs (four unigenes) were annotated as *LIM domain *and four ESTs (one unigene) as *MADS-box*. A tobacco LIM protein can bind to the Pal-box sequence in the promoter regions of several phenylpropanoid biosynthesis genes [61]. Some *Arabidopsis MADS-box *genes have been implicated in the regulation of lignin biosynthesis [62].

### Gene expression and wood properties

Wood formation is a complex and continuous process involving a series of developmental stages during which considerable variation in wood properties is observed. Compared to mature wood, juvenile wood shows higher spiral grain, lower density, higher microfibril angle (MFA), more longitudinal shrinkage, lower cellulose content, higher lignin content, more pectin and shorter tracheids [1,3,74]. Compared to latewood, earlywood has higher growth rate, thinner cell walls, larger radial lumen, more lignin, lower hemicellulose, lower pectin, slightly lower cellulose, higher MFA and lower density [74-76].

**Figure 5:**
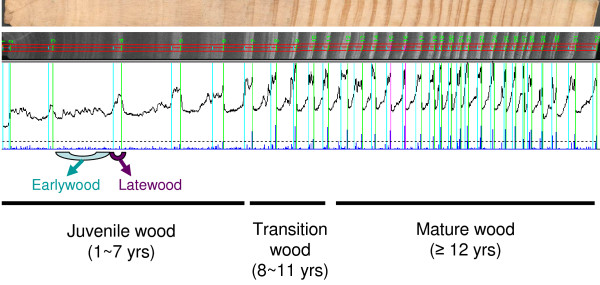
**A wood core and SilviScan profile of wood density for a 32-year-old radiata pine tree**. The cross section of wood core sampled at 1.3 m height shows the annual change in width and colour of earlywood and latewood within each ring. Wood density is the most important wood trait for the sawmill timber industry. SilviScan profiling for wood density of radiata pine revealed the distinct characteristics of juvenile wood, particularly juvenile earlywood, compared to transition and mature wood. SilviScan analysis was performed in the method of Evans, et al, 2000 [78].

Most of the distinguishing properties of juvenile wood are due to the high proportion of earlywood in the juvenile wood [77]. The SilviScan profiling [78] of mature radiata pine trees revealed the unique morphology and wood properties of juvenile earlywood compared to other wood development stages (Figure 5 and Additional file 6). Juvenile earlywood forms a larger proportion in the early rings and has lower density, higher microfibril angle (MFA), lower modulus of elasticity (MOE) and thinner cell walls. These unique phenotypic characteristics of juvenile earlywood are likely to be caused by its distinct transcriptome (Figure 3A, B). Some genes involved in cellulose synthesis and secondary wall formation are more strongly expressed in latewood and mature wood than in earlywood and juvenile wood, respectively (P-value < 0.05) (Additional file 3 and 4), which may be associated with some of the observed differences in wood quality between the different wood tissues, such as cellulose content and cell wall thickness. Many genes related to lignin biosynthesis are more abundant in earlywood and mature wood than in latewood and juvenile wood, respectively; however, only some genes are statistically significant (P-value < 0.05) (Table 7). These differences in gene abundance may give rise to the higher lignin content found in earlywood and mature wood.

**Table 7:** Comparisons of EST abundance of genes related to lignin biosynthesis in different wood development stages.

*Gene*	*EST abundance in EW and LW*			*EST abundance in JW and MW*		
		
	EW ^a^	LW^b^	P-value ^e^	JW ^c^	MW ^d^	P-value ^e^
*CCoAOMT*	16	13	> 0.05	9	12	0.04
*C*4*H*	10	8	> 0.05	4	4	> 0.05
*4CL*	10	6	> 0.05	8	5	> 0.05
*CAD*	6	3	0.07	1	4	0.04
*C*3*H*	7	2	> 0.05	2	3	> 0.05
*COMT*	2	6	> 0.05	3	3	> 0.05
*CCR*	1	1	> 0.05	2	0	> 0.05
*PAL*	0	2	> 0.05	2	0	> 0.05
*Peroxidase*	11	5	0.08	0	7	0.001
*Laccase*	16	20	> 0.05	16	10	> 0.05
*SAMS*	15	31	0.05	19	11	> 0.05
*Dirigent-like*	4	3	> 0.05	3	1	> 0.05
*Cytochrome c oxidase*	7	7	> 0.05	10	0	0.01

^a^: The number of ESTs for the combined earlywood (EW) libraries. Total ESTs in the combined earlywood libraries from juvenile, transition and mature age are 2,790;

^b^: The number of ESTs for the combined latewood (LW) libraries. Total ESTs in the combined latewood libraries from juvenile, transition and mature age are 3,169;

^c^: The number of ESTs for the combined juvenile wood (JW) libraries. Total ESTs in the combined JW libraries from earlywood and latewood tissues are 2,902;

^d^: The number of ESTs for the combined mature wood (MW) libraries. Total ESTs in the combined MW libraries from earlywood and latewood tissues are 1,564;

^e^: Statistical significance according to Audic and Claverie 1997 [79].

Although Audic and Claverei proposed a statistical method [79] to test the significance of digital gene expression, the preferentially expressed genes (even statistically significant genes) derived from gene abundance analysis still require further validation, such as microarray experiments, real time RT-PCR or other alternative methods. Multiplex ligation-dependent probe amplification (MLPA) [80] was used in this study for validation of selected genes (Table 8). The normalized ratio of EST abundance and MLPA gene expression ratio is highly comparable for a *proline-rich protein *(*PRP*) gene in both earlywood and latewood at transition age, as well as for a *dehydrin *gene in latewood at transition age. The MLPA gene expressions of *AGP4 *and *peroxidase *at both juvenile and transition ages confirmed their high EST abundances in earlywood. However, MLPA expression values of some genes may differ from their EST abundances (Table 8), suggesting EST abundance could not infer the exact gene expression level.

**Table 8:** RT-MLPA validation of four genes preferentially represented in earlywood and latewood.

*Gene*	*EST abundance*			*MLPA ratio*			
		
	EW/LW	Normalized ratio	P-value	JE/JL *	P-value	TE/TL **	P-value
*AGP4*	34/11	3.51	0.001	1.36	< 0.001	13.5	< 0.001
*Peroxidase*	11/1	12.49	0.001	2.59	< 0.001	5.74	< 0.001
*Proline-rich protein (PRP)*	13/3	4.92	0.005			4.33	< 0.001
*Dehydrin*	2/17	0.13	0.001			0.55	< 0.001

*: JE, juvenile earlywood; JL, juvenile latewood;

**: TE, transition earlywood; TL, transition latewood.

## Conclusion

The radiata pine genomic resource was developed from six developing xylem libraries with 6,389 high quality ESTs and 3,304 unigenes. This is the first large scale and publicly available genomic resource for radiata pine. Many genes involved in cell wall biosynthesis and transcription factors were identified in the wood formation of radiata pine. Comparative analysis among different development stages revealed a distinct transcriptome in juvenile earlywood. Genes with relatively more expression in earlywood, latewood, juvenile wood and mature wood were also identified, respectively. The identified genes in this study will be candidates for functional genomics and association studies in radiata pine. This genomic resource will also be valuable for comparative genomics of wood formation in forest trees.

## Methods

### Plant material

In order to broadly represent the xylogenesis transcriptome in radiata pine, developing xylem tissues were sampled at six critical development stages: earlywood (spring) and latewood (autumn) tissues collected at juvenile (7 yrs), transition (11 yrs) and mature (30 yrs) ages. Two 7-year-old and two 30-year-old trees were sampled at Yarralumla ACT in August 2004 (spring) and April 2005 (autumn). Three 11-year-old trees were sampled at Bondo NSW in September 2003 (spring) and April 2004 (autumn). Developing xylem tissues were collected by scraping the thin (approximately 1.0 mm) and partially lignified layer on the exposed xylem surface at breast height. All xylem tissues were immediately frozen in liquid nitrogen in the field, and then stored in the laboratory at -80°C for later RNA extraction.

### cDNA library construction and colony isolation

Total RNA was extracted using the method of Chang, et al [81] with slight modification. Poly(A)^+ ^mRNA was isolated using the polyATtract system III/IV (Promega, CA). Five micrograms of mRNA were used as starting template for cDNA library construction using the ZAP-cDNAGigapack III Gold Cloning Kit (Stratagene, CA). After first and second strand cDNA synthesis, the radioactive cDNA samples were checked on an alkaline agarose gel and exposed to x-ray film. The double strand cDNAs were fractionated using a drip column filled with Sepharose CL-2B gel. The fractions at approximately 600 bp or more were pooled and ligated into the EcoRI/XhoI cloning site in the Uni-ZAP XR vector. One to two microliters of ligated cDNA inserts were packaged using Gigapack III Gold Packaging Extract. To make a large quantity of high titer stock of the lambda phage library, the primary libraries were amplified according to the manufacturer's protocols (Stratagene, CA). The pBluescript phagemids containing cDNA inserts were mass excised in vivo from the Uni-ZAP XR vector using Ex-Assist helper phage and XL1-Blue MRF'. The titer of lambda phage and phagemid libraries was measured using the host cells of XL1-Blue MRF' strain and SOLR strain, respectively. Library quality and the size of cDNA inserts were determined by PCR screening of 96 randomly selected clones. All lambda phage cDNA libraries were stored at -80°C.

The excised phagemids were replicated in SOLR cells and grown on LB-ampicillin agar plates overnight at 37°C for colony isolation. All phagemid colonies were picked out and put into LB broth with ampicillin. Colony picking was done by a Versarray Colony Picking Robot (Bio-Rad, CA) or manually with a tooth-pick. The picked colonies were cultured overnight at 37°C with shaking. These suspension cultures were used for PCR reactions and EST sequencing, or stored at -80°C in glycerol stock for later use.

### EST sequencing and assembly

An average of 1,200 cDNA clones from each of the six libraries was used for EST sequencing. Approximately 8,000 sequencing reactions were performed on a total of 7,200 cDNA clones. The cDNA inserts were PCR amplified using the M13 forward and reverse primers followed by clean-up with ExoSap (GE Healthcare, UK). The sequencing primer for the 5' end of cDNA inserts was the SK, T3 or M13 primer. A small proportion of sequences were obtained from the 3' end using the M13 forward primer. The sequencing reaction was performed using BigDye 3.1. After a quick start at 96°C 1 min, the reaction was cycled 25 times at 96°C 1 sec, 50°C 5 sec and 60°C 4 min, followed by holding at 4°C. After sodium acetate-EDTA-ethanol precipitation, the sequencing reaction was run using ABI 3730 capillary sequencers (Applied Biosystems, CA). The trace files were base called by Sequencing Analysis v5.2 which was integrated into the ABI 3730 capillary sequencers.

The raw EST sequences were processed using Sequencher 4.7 (Gene Codes Corporation, MI) to trim vector and ambiguous ends. The poly(A) in the EST sequences was deleted manually. A total of 6,389 high quality ESTs with at least 100 bp in length were obtained. These ESTs were submitted to dbEST at the National Center for Biotechnology Information with Genbank accession numbers FE518213 to FE524601. The 5' end ESTs from 5,952 different clones were assembled to generate consensus sequences for unigenes, including contigs and singletons. The EST assembly was performed using the CAP3 program [82] integrated in BIO301, an automated EST sequence management and functional annotation system [83]. In order to minimize chimeric contigs in assembly, an overlap of at least 40 bp and identity of sequence of at least 95% were used as thresholds in the assembly. The number of ESTs in each contig was used to estimate the redundancy in the EST sequencing.

### Annotation and functional classification

The high quality ESTs and derived unigenes were used to search the NCBI nr (non-redundant) database using blastx and tblastx (E-value ≤ 10^-5^). They were also used to search the UniProt known protein database [84] using blastx (E-value ≤ 10^-5^). The sequences with no matches in UniProt were further blasted against the TIGR all tentative consensus sequence gene indices database [85] using blastn (E-value ≤ 10^-15^). Putative functions for ESTs and unigenes were assigned with GO (Gene Ontology) terms [86]. To identify the known protein families, the ESTs were also searched with the Pfam protein family database [87] using blastx (E-value ≤ 10^-5^). Initial functional classification was based on the assigned GO terms with two levels of classification. Gene enrichment analysis and further functional classification on GO terms were conducted using the DAVID Bioinformatics Resources 2008 [23,24]

### Identification and validation of differentially represented genes

Comparison of EST abundance was used to identify differentially represented genes in different libraries or combined libraries. Number of ESTs for each gene in different libraries was previously normalized at the presumed same number of ESTs in each library. A normalized ratio of EST abundance was calculated for the comparisons. The significance of differentially represented genes was statistically tested using the method from Audic and Claverie [79].

Preferentially represented genes were validated using the strategy of multiplex ligation-dependent probe amplification (MLPA) [80]. More specifically we used the RT-MLPA protocol for mRNA detection and quantification. Four genes (*AGP4*, *proline-rich protein*, *peroxidase *and *dehydrin*) preferentially represented in the combined earlywood or latewood libraries were included in the validation. Gene expressions of these four genes were detected in earlywood and latewood tissues from three radiata pine trees at transition age (11 yrs). In addition, expressions of *AGP4 *and *peroxidase *were also measured in earlywood and latewood tissues from eight radiata pine trees at juvenile age (5 yrs). We used four technical replicates in RT-MLPA. Reverse transcription for ~400 ng total RNA after DNase treatment was performed using the ImProm-II Reverse Transcription System (Promega, WI) and oligo (dT)_15_. Synthesized cDNA was hybridized with mixed NPK and LIG probes designed for the validated genes (Additional file 7) at 60°C overnight, followed by ligation and PCR amplification with SALSA D4 primer. Mixture PCR products from multiple genes were separated and analysed using the CEQ™ 8000 Genetic Analysis System (Beckman Coulter, CA).

## Abbreviations

Mbp: million base pairs; EST: expressed sequence tag; E-value: expected-value; GO: gene ontology; *CesA*: *cellulose synthase*; *4CL*: *4-cinnamoyl CoA ligase*; *C3H*: *p-coumarate 3-hydroxylase*; *C4H*: *cinnamate 4-hydroxylase*; *CAD*: *cinnamyl alcohol dehydrogenase*; *CCoAOMT*: *caffeoyl CoA O-methyltransferase*; *CCR*: *cinnamoyl CoA reductase*; *COMT*: *caffeic acid O-methyltransferase*; *PAL*: *phenylalanine ammonia-lyase*; *SAMS: S-adenosylmethionine synthetase*; *SuSy*: *sucrose synthase*; *MetE*: *methionine synthase *(*cobalamin-independent*); *TUB*: *tubulin*; AGP: arabinogalactan protein; FLA: fasciclin-like arabinogalactan protein; MIP: major intrinsic protein; PRP: proline-rich protein; LP: water deficit inducible protein; SNPs: single nucleotide polymorphisms; TF: transcription factor; MFA: microfibril angle; JW: juvenile wood; TW: transition wood; MW: mature wood; EW: earlywood; LW: latewood; JE: juvenile earlywood; JL: juvenile latewood; TE: transition earlywood; TL: transition latewood; ME: mature earlywood; ML: mature latewood; MLPA: multiplex ligation-dependent probe amplification; RT-MLPA: reverse transcriptase MLPA.

## Authors' contributions

XL carried out cDNA library construction, EST sequencing, assembly, annotation, functional classification, gene identification and manuscript preparation. SS and HW proposed the research project and guided the research. SD participated in colony isolation and PCR. All the authors have read and approved the final manuscript.

## Supplementary Material

Additional file 1 **Functional classification of 2,101 unigenes with homologs in UniProt database.** The 16 functional groups with Enrichment Scores 0.11–4.52 for the 2,101 radiata pine unigenes, which have homologs in UniProt database, were revealed with DAVID functional classification. Click here for file

Additional file 2 **Identification of cell wall related genes in the radiata pine EST resource.** The table represents 18 categories and 91 families of cell wall related genes based on the MAIZEWALL database. Click here for file

Additional file 3 **Protein families more abundant in combined earlywood, latewood, juvenile wood and mature wood.** The table listed 15, 19, 5 and 29 protein families more abundant in combined earlywood, latewood, juvenile wood and mature wood, respectively. Most of the listed protein families are statistically significant based on P-value. Click here for file

Additional file 4 **Genes or gene families more strongly expressed in combined earlywood, latewood, juvenile wood and mature wood.** The table listed 20, 18, 20 and 25 genes or gene families more strongly expressed in combined earlywood, latewood, juvenile wood and mature wood, respectively. Most of the listed genes or gene families are statistically significant based on P-value. Click here for file

Additional file 5 **Specifically enriched GO terms in earlywood, latewood, juvenile wood and mature wood.** The table provided 16, 18, 21 and 11 GO terms specifically enriched in earlywood, latewood, juvenile wood and mature wood, respectively. Most of the listed GO terms are statistically significant based on P-value. Click here for file

Additional file 6 **Wood quality variation in different wood tissues of radiata pine.** The table shows wood quality variation among different wood tissues of radiata pine. The nine wood traits were measured on 10 trees of 25-year-old using SilviScan profiling. Click here for file

Additional file 7 **NPK- and LIG- probes for RT-MLPA validation.** The table listed NPK- and LIG- probes used in RT-MLPA validation for four genes (*dehydrin*, *PRP*, *peroxidase *and *AGP4*), which are preferentially represented in earlywood or latewood tissues at juvenile or transition ages. Click here for file
